# Role of Venoarterial Extracorporeal Membrane Oxygenation as a Procedural Support Tool in Cardiovascular Instability: A Case Series

**DOI:** 10.1155/cric/9598287

**Published:** 2025-12-29

**Authors:** Seonghyeon Bu

**Affiliations:** ^1^ Division of Cardiology, Department of Internal Medicine, Uijeongbu St. Mary’s Hospital, College of Medicine, The Catholic University of Korea, Seoul, Republic of Korea, catholic.ac.kr; ^2^ Catholic Research Institute for Intractable Cardiovascular Disease (CRID), College of Medicine, The Catholic University of Korea, Seoul, Republic of Korea, catholic.ac.kr

**Keywords:** cardiac arrest, cardiogenic shock, ECLS shock, myocardial infarction, VA-ECMO

## Abstract

The use of venoarterial extracorporeal membrane oxygenation (VA‐ECMO) is increasing worldwide. Patients with cardiogenic shock were the first indication for VA‐ECMO according to the Extracorporeal Life Support Organization (ELSO) guidelines. However, recent studies have shown that VA‐ECMO is not beneficial in patients with cardiogenic shock. This report describes the case of three patients who presented with infarct‐related cardiac arrest without cardiogenic shock and received VA‐ECMO to support coronary intervention. These patients tolerated the treatment and showed a good prognosis at discharge. These cases describe the potential role of VA‐ECMO as a supportive intervention in patients with infarct‐related cardiac arrest without cardiogenic shock.

## 1. Introduction

In patients with cardiogenic shock, the use of venoarterial extracorporeal membrane oxygenation (VA‐ECMO) has gradually increased despite limited evidence. In South Korea, annual trends in the number of ECMO cases showed a 170‐fold increase, from 14 in 2004 to 2387 in 2017, with the most common cause of ECMO being cardiac failure (approximately two‐thirds). The mortality rate did not change significantly (approximately 60%) [1]. According to the ELSO guidelines, the major indication for VA‐ECMO is cardiogenic shock, and the presenting situations are medical (such as acute myocardial infarction, fulminant myocarditis, and massive pulmonary embolism) and postsurgical situations (such as postcardiotomy shock and posttransplantation shock) [2]. However, recent studies have shown that VA‐ECMO is not beneficial in patients with infarct‐related cardiogenic shock. In the ECMO‐CS trial (Extracorporeal Membrane Oxygenation in the Therapy of Cardiogenic Shock), immediate implementation of VA‐ECMO did not improve outcomes compared with no immediate VA‐ECMO in patients with severe or rapidly deteriorating cardiogenic shock. A large proportion (39%) of patients in the no early VA‐ECMO group subsequently received VA‐ECMO or other mechanical circulatory support due to further hemodynamic deterioration [3]. The ECLS‐SHOCK trial also showed that all‐cause death at 30 days was not lower in patients who received VA‐ECMO than in those who received medical therapy alone. Cardiogenic shock was defined as a systolic blood pressure < 90 mmHg, lactate level > 3 mmol/L, and signs of impaired organ perfusion [4]. While VA‐ECMO provides full cardiopulmonary support, recent randomized data indicate no mortality reduction with early, unselected use in infarct‐related cardiogenic shock and highlight higher complication rates. There is little evidence regarding the use of VA‐ECMO in patients with cardiogenic shock, and the role of VA‐ECMO remains unclear. Accordingly, the present report focuses on a narrower context—infarct‐related cardiac arrest without established cardiogenic shock—in which short‐term VA‐ECMO may serve as a bridge to reperfusion in carefully selected patients. Herein, we present the cases of three patients who showed a good prognosis with infarct‐related cardiac arrest without cardiogenic shock using VA‐ECMO.

## 2. Case Presentation

### 2.1. Patient 1

**Figure 1:**
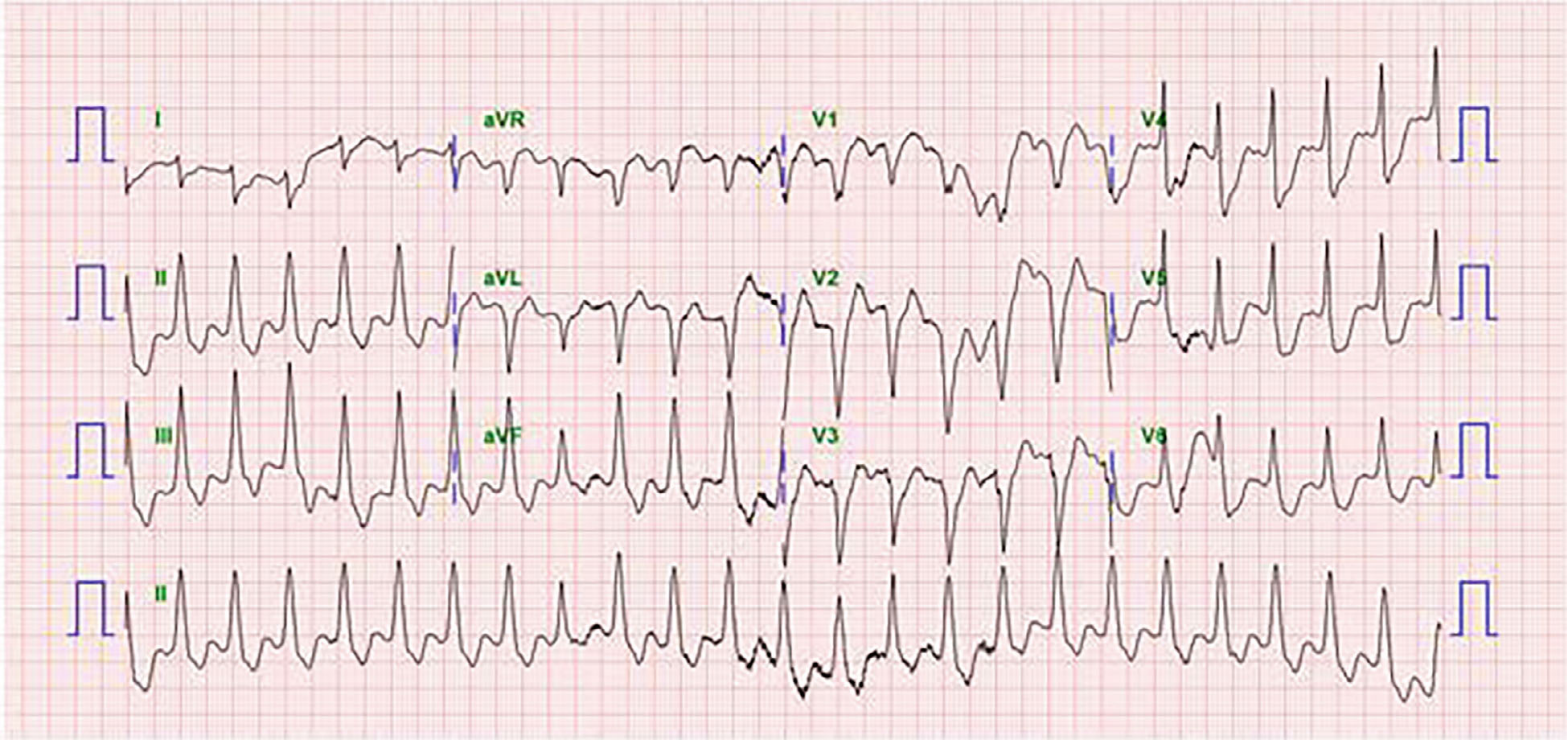
Ventricular tachycardia.

A 66‐year‐old male visited the emergency room after the return of spontaneous circulation. The patient collapsed while lifting heavy loads, and a bystander performed cardiopulmonary resuscitation (CPR). His medical history included hypertension and Type 2 diabetes mellitus. Initial mental status was confused, and vital signs were as follows: blood pressure, 175/137 mmHg; heart rate, 145 bpm; and oxygen saturation, 95% on room air. Electrocardiography (ECG) revealed ventricular tachycardia (Figure 1). DC cardioversion was performed, and emergent coronary angiography (CAG) was planned. Within 1 h, the patient was moved to the catheterization laboratory. Before CAG, the patient collapsed again. CPR was initiated, and ECMO was instituted. The ECMO system used was Capiox EBS (Terumo Ltd., Tokyo, Japan). A 17‐Fr cannula was inserted into the left femoral artery and a 21‐Fr cannula into the right femoral vein using the Seldinger method. Before cannulation, 3000 IU of unfractionated heparin was administered, and the activated clotting time (ACT) was maintained within 160–180 s. The initial blood flow was 2.73 L/min/m^2^, and the pump speed was 1932 rpm. The total ischemic time was 14 min. CAG revealed that the middle right coronary artery (RCA) was totally occluded, and percutaneous coronary intervention (PCI) was successfully performed (Figure 2). Systemic perfusion recovery was assessed by a lactate level below 5 mmol/L together with improvement on chest radiography, and cardiac function recovery was assessed by a left ventricular outflow tract velocity–time integral (LVOT VTI) greater than 10 cm and a left ventricular ejection fraction (LVEF) greater than 20%. To assess hemodynamic stability under minimal support, VA‐ECMO blood flow was reduced to very low assistance (1.0 L/min/m^2^), and it was confirmed that the mean arterial pressure (MAP) remained above 60 mmHg in the absence of low‐dose inotropes. On Day 7, the patient was weaned off ECMO. Transthoracic echocardiography (TTE) revealed an LVEF of 46% with an ischemic insult to the RCA territory. ECG revealed atrial fibrillation. The patient stabilized and was discharged on Day 13. The discharge medications included aspirin 100 mg qd, clopidogrel 75 mg qd, edoxaban 30 mg qd, rosuvastatin 20 mg qd, ezetimibe 10 mg qd, carvedilol 3.125 mg bid, amiodarone 100 mg qd, and empagliflozin 10 mg qd.

Figure 2Totally occluded right coronary artery (a) and successful percutaneous coronary intervention (b).

**Figure:**
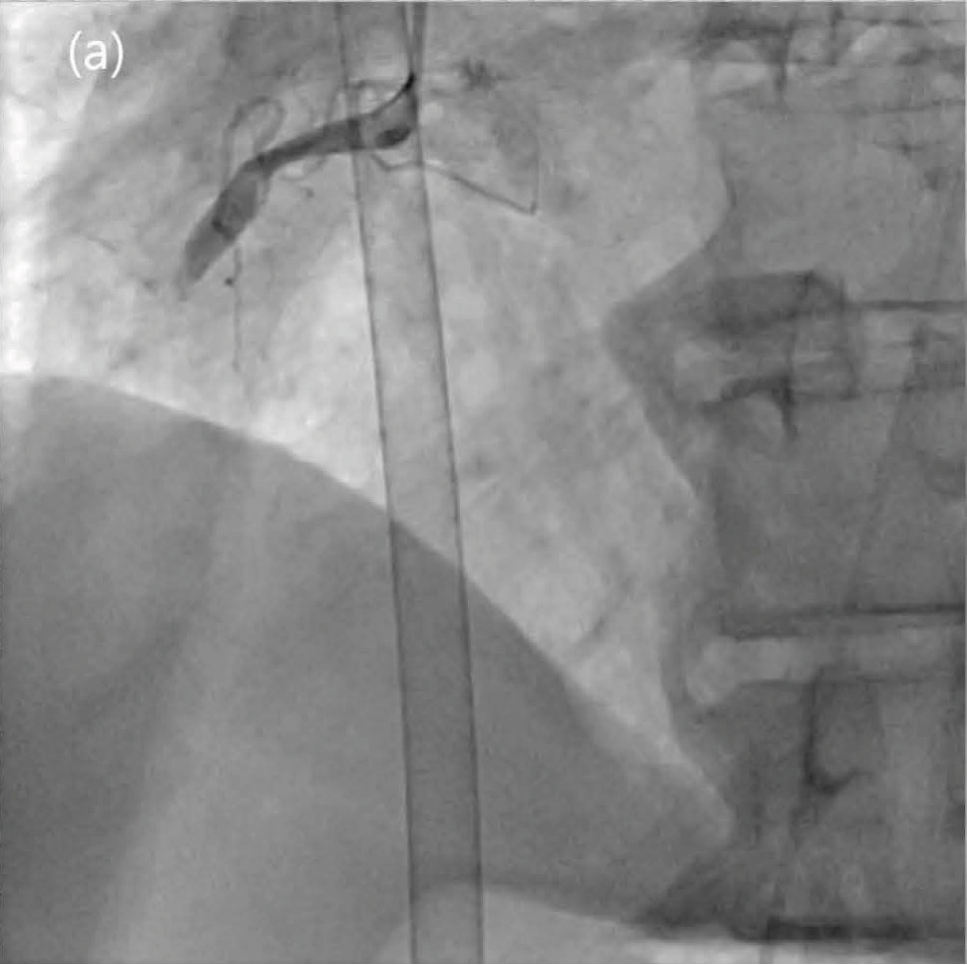
(a)

**Figure:**
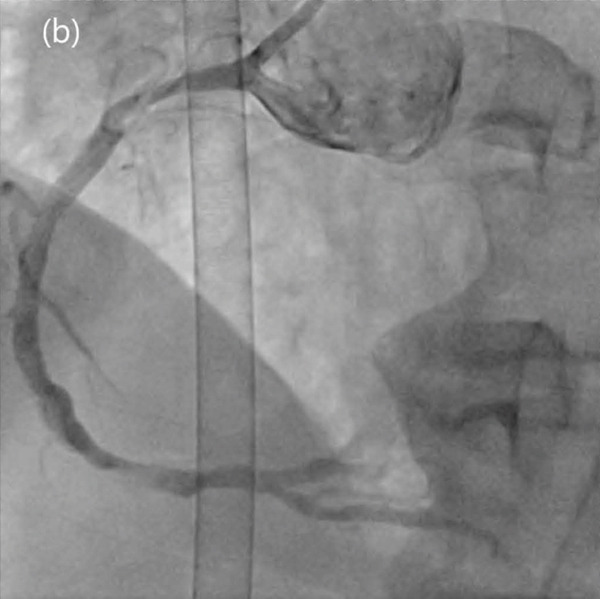
(b)

### 2.2. Patient 2

A 78‐year‐old male visited the clinic complaining of chest pain persisting for 3 months and pain that was aggravated due to cold winds for several days. The patient had chronic kidney disease. His blood pressure was 118/78 mmHg, and his heart rate was 112 bpm. The physical examination results were unremarkable. ECG revealed a regular sinus rhythm without ST‐segment changes. TTE revealed an LVEF of 46% with global hypokinesia of the left ventricle (LV) wall. The patient presented with symptoms characterized by a sensation of heaviness, more frequently triggered in cold weather and subsiding within 1–5 min after discontinuation of effort, suggesting a moderate clinical likelihood of obstructive coronary artery disease. CAG was performed in the presence of LV systolic dysfunction and the need for symptom control. Elective CAG showed significant stenosis of the left main (LM) artery, and PCI was initiated. With regard to the choice between PCI and coronary artery bypass graft (CABG), the surgical risk was low, with an STS score of 2.83%, and the anatomical risk was also low, with a SYNTAX score of 22. Because complete revascularization is feasible with PCI, PCI was chosen over CABG. The double‐kissing crushing technique was performed; however, thrombus formation in the LM occurred after the final kissing balloon inflation. Subsequently, no reflow was observed in the LM, leading to cardiac arrest. During bifurcation PCI, thrombus formation may result from plaque rupture or tissue protrusion with distal microembolization of atherothrombotic debris and may also be related to stent underexpansion or malapposition. CPR was initiated, and ECMO was instituted during the ensuing 14 min of ischemia. The ECMO system used was Capiox EBS (Terumo Ltd., Tokyo, Japan). A 17‐Fr cannula was inserted into the left femoral artery and a 21‐Fr cannula into the left femoral vein using the Seldinger method. Before cannulation, 3000 IU of unfractionated heparin was administered, and the ACT was maintained within 160–180 s. The initial blood flow was 2.67 L/min/m^2^, and the pump speed was 1525 rpm. Thrombus aspiration, additional final kissing ballooning, and proximal optimization were performed, and the procedure was completed with thrombolysis in myocardial infarction (TIMI) 3 flow (Figure 3). ECMO weaning was performed according to the same criteria described for Patient 1. On Day 7, the patient was weaned off ECMO, and TTE showed an LVEF of 35%, which was slightly lower than the LVEF before, with a newly noted ischemic insult in the left anterior descending (LAD) and left circumflex (LCX) territories. The patient stabilized and was discharged on Day 15. The discharge medicines were as follows: aspirin 100 mg qd, clopidogrel 75 mg qd, rosuvastatin 20 mg qd, ezetimibe 10 mg qd, carvedilol 6.25 mg bid, sacubitril/valsartan 50 mg bid, dapagliflozin 10 mg qd, spironolactone 12.5 mg qd, and nicorandil 5 mg bid.

Figure 3Thrombus formation in the left main (LM) artery after final kissing ballooning (a), followed by totally occluded LM (b). Final coronary angiogram showing thrombolysis in myocardial infarction 3 flow (c).

**Figure:**
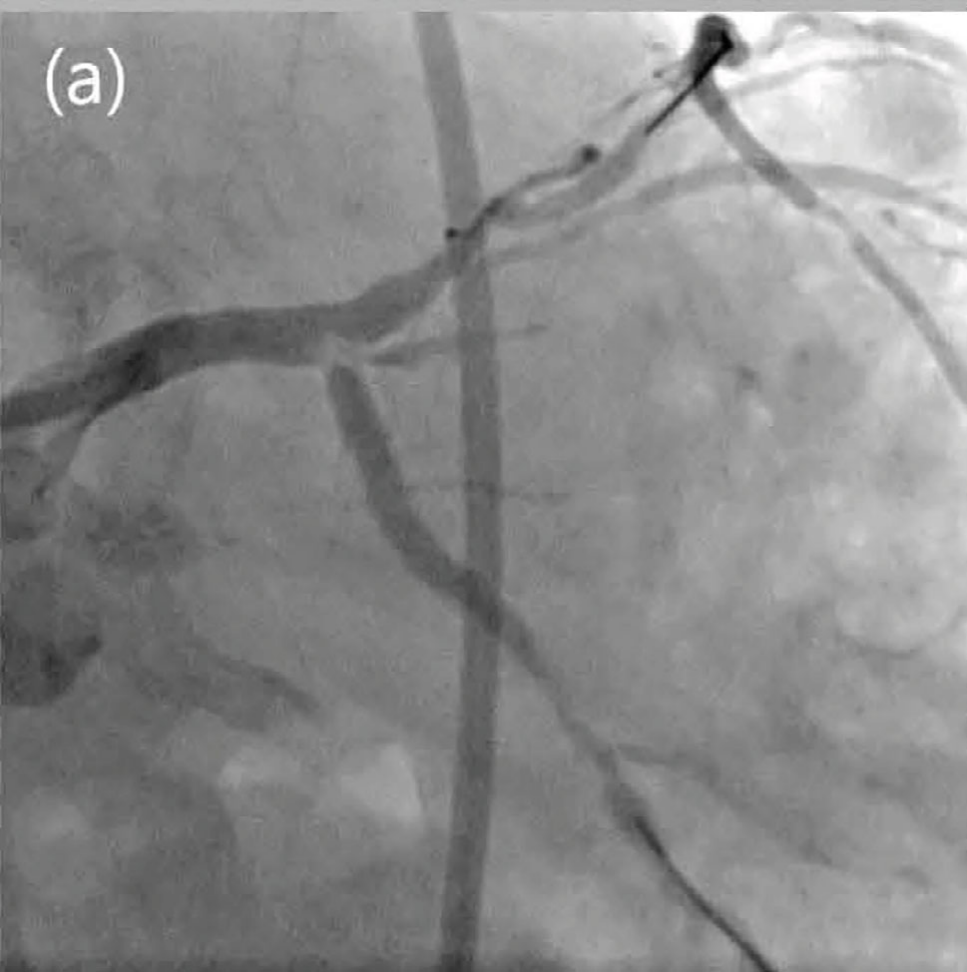
(a)

**Figure:**
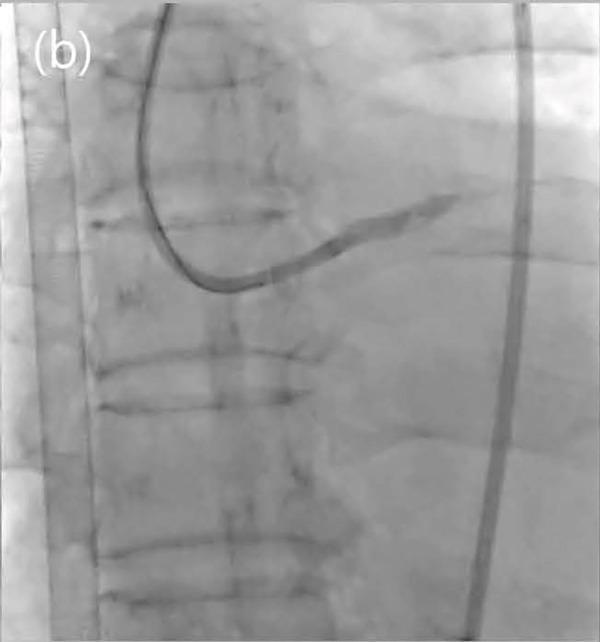
(b)

**Figure:**
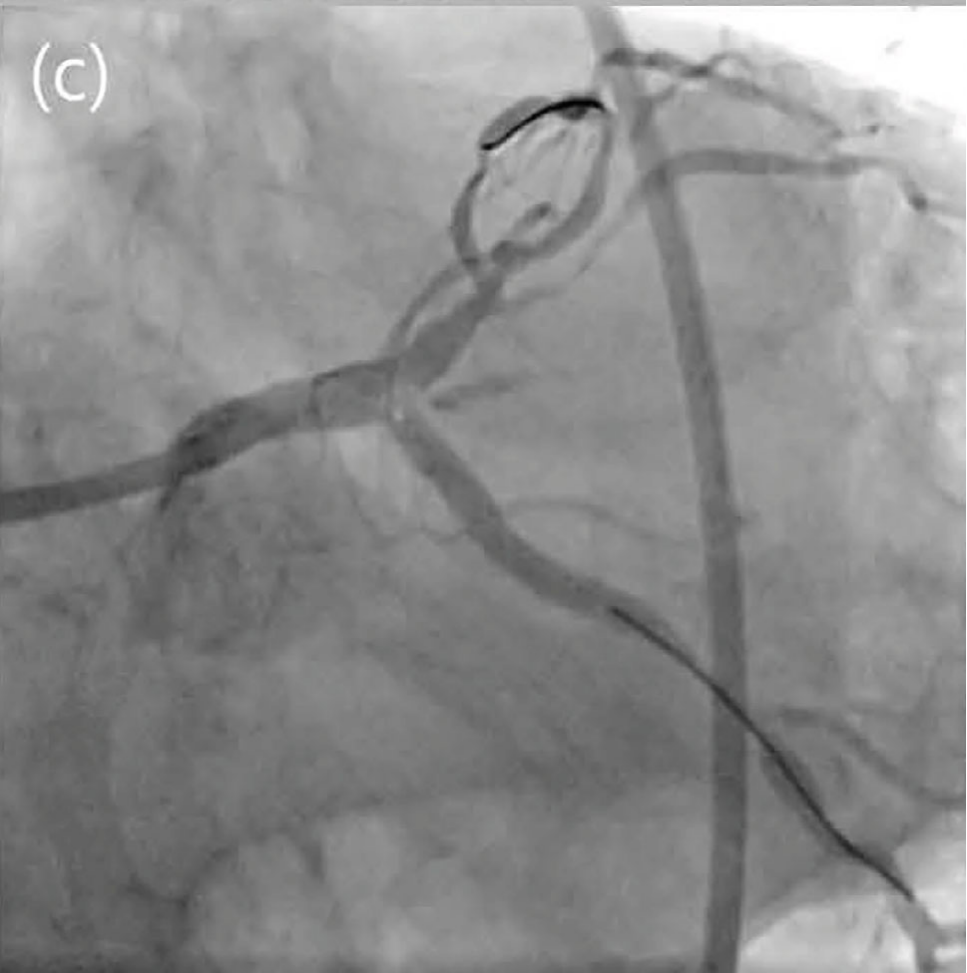
(c)

### 2.3. Patient 3

A 46‐year‐old male visited the emergency room because of an altered mental status after collapsing during heavy lifting. During transportation by the emergency medical service team, defibrillation was performed because of pulseless ventricular tachycardia, and the patient′s mental state at admission was confused. His blood pressure was 92/63 mmHg, and his heart rate was 97 bpm. The ECG revealed ST‐segment depression in the diffuse leads and elevation in the aVR lead. Emergency CAG showed that the distal LM was completely occluded, and onsite PCI was successfully performed (Figure 4). However, the patient collapsed due to reperfusion injury, and CPR was initiated. ECMO was inserted during an ischemic period of 9 min. The ECMO system used was Capiox EBS (Terumo Ltd., Tokyo, Japan). A 17‐Fr cannula was inserted into the left femoral artery and a 21‐Fr cannula into the left femoral vein using the Seldinger method. Before cannulation, 3000 IU of unfractionated heparin was administered, and the ACT was maintained within 160–180 s. The initial blood flow was 3.39 L/min/m^2^, and the pump speed was 1925 rpm. The patient was transferred to the intensive care unit and gradually recovered. ECMO weaning was performed according to the same criteria described for Patient 1. On Day 10, the patient was weaned off ECMO, and TTE showed an LVEF of 38% with ischemic insult of the LAD territory. The patient stabilized and was discharged on Day 16. The discharge medications included aspirin 100 mg qd, prasugrel 10 mg qd, atorvastatin 80 mg qd, and colchicine 0.6 mg qd. Medicines for heart failure with reduced ejection fraction were gradually added later during the outpatient clinic visits because the blood pressure was low at 107/58 mmHg.

Figure 4Totally occluded left main artery (a) and successful reperfusion (b).

**Figure:**
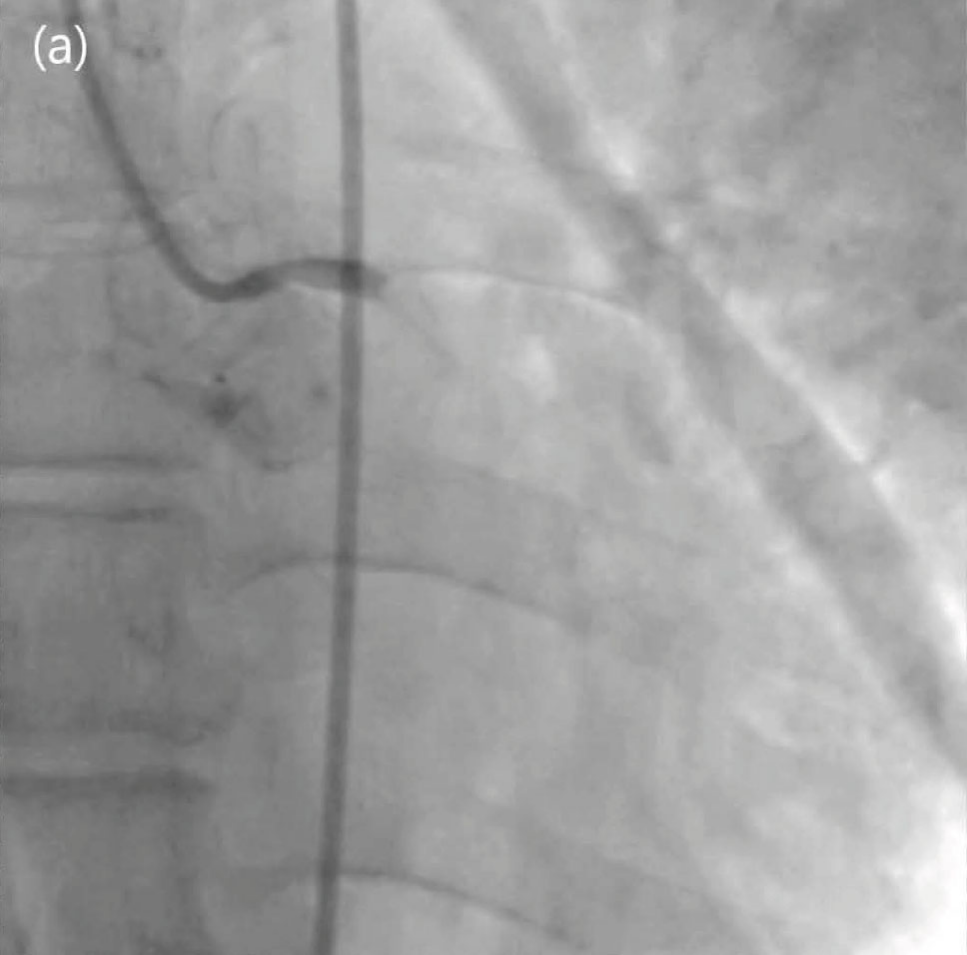
(a)

**Figure:**
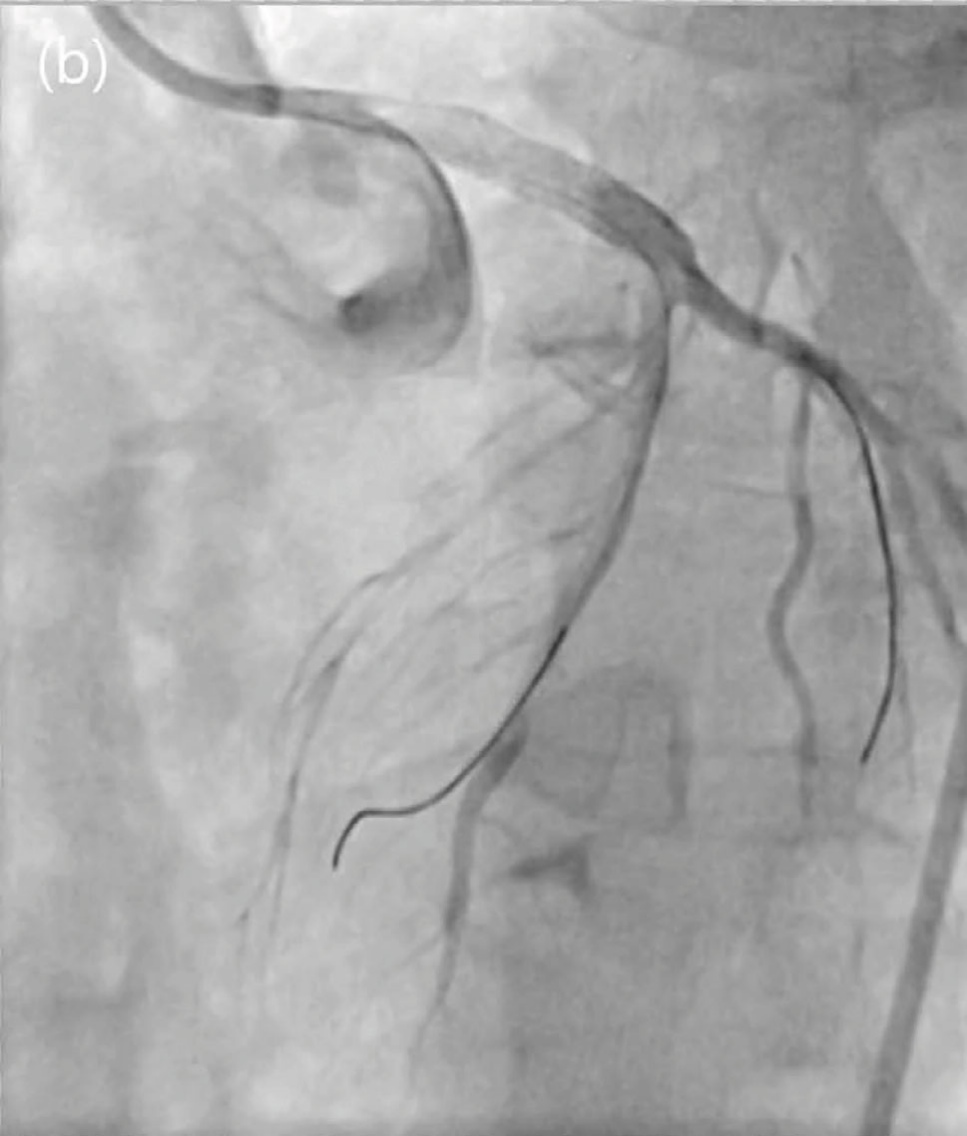
(b)

## 3. Discussion

**Figure 5:**
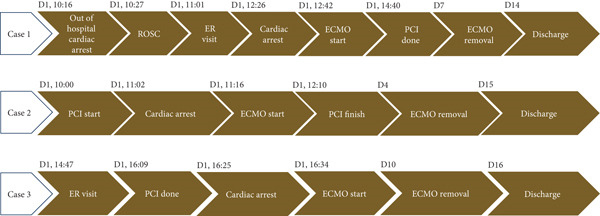
The clinical timeline for three patients. ECMO, extracorporeal membrane oxygenation; ER, emergency room; PCI, percutaneous coronary intervention; ROSC, return of spontaneous circulation.

These three cases showed that patients with infarct‐related cardiac arrest without cardiogenic shock recovered significantly within a few days of VA‐ECMO use. Patient 1 presented with ST elevation myocardial infarction, and the culprit lesion was the RCA. The initial blood pressure was 175/137 mmHg, which did not indicate cardiogenic shock. The patient experienced recurrent collapse before coronary revascularization; therefore, VA‐ECMO was instituted to provide support and allow completion of the procedure. Patient 2 presented with unstable angina, and the culprit lesion was located at the LM bifurcation. The initial blood pressure was 118/78 mmHg. The patient collapsed during coronary revascularization because of plaque rupture following total occlusion of the LM coronary artery. VA‐ECMO was immediately performed, and coronary revascularization was successfully completed. Patient 3 presented with ST elevation myocardial infarction, and the culprit lesion was a completely occluded LM coronary artery. His blood pressure was 92/63 mm Hg, which was not indicative of cardiogenic shock. Coronary revascularization was rapidly completed, resulting in TIMI 3 coronary flow. However, the patient collapsed after revascularization due to reperfusion injury, a complication that can occur after coronary reperfusion in the setting of extensive cardiomyocyte damage or necrosis [5, 6]. VA‐ECMO was performed, and the patient gradually recovered. The clinical timeline of these patients is shown in Figure 5. The common features among the three patients were as follows: (A) initial systolic blood pressure was not in the shock range (> 90 mmHg), and (B) the indication for VA‐ECMO was infarct‐related cardiac arrest. The role of VA‐ECMO in this setting is to provide circulatory support during coronary revascularization in patients with infarct‐related cardiac arrest but without cardiogenic shock.

The ELSO guidelines for VA‐ECMO recommend considering it in patients with refractory cardiogenic shock due to a correctable cause [2]. Cardiogenic shock suitable for VA‐ECMO is described by a systolic blood pressure less than 90 mmHg, urine output < 30 mL/h, lactate level > 2 mmol/L, and an altered consciousness state. However, the ECLS‐SHOCK trial, which used a similar definition of cardiogenic shock, showed that all‐cause mortality at 30 days was not lower in patients with infarct‐related cardiogenic shock who received VA‐ECMO than in those who received medical therapy. Subgroups, including sex, old age, diabetes, cardiac arrest, and lactate levels, showed no significant benefit for any cause of death at 30 days [4]. An individual patient data meta‐analysis, including the ECLS‐SHOCK trial, also showed that VA‐ECMO did not reduce all‐cause death at 30 days compared with medical therapy [7]. The ECMO‐CS trial was an open‐label, randomized controlled study that compared immediate VA‐ECMO with early conservative therapy in patients with cardiogenic shock, approximately 65% of whom had myocardial infarction. The trial showed that VA‐ECMO did not provide superior outcomes compared with initial conservative management for the primary composite endpoint of all‐cause mortality, implantation of another mechanical circulatory support device, or resuscitated cardiac arrest. Although the severity of cardiogenic shock was relatively modest, defined by a systolic blood pressure of < 100 mmHg, and the crossover rate between groups reached 39%, no subgroup analysis revealed a beneficial effect of VA‐ECMO [3].

**Figure 6:**
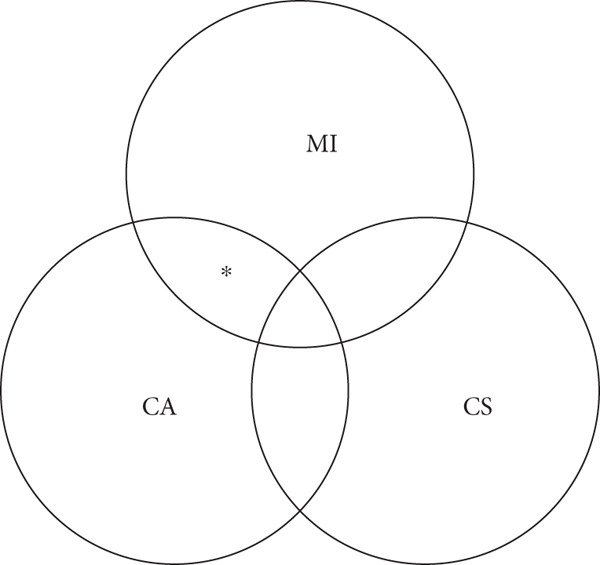
Selected patients with infarct‐related cardiac arrest without cardiogenic shock. MI, myocardial infarction; CA, cardiac arrest; CS, cardiogenic shock.

Recent studies have shown that VA‐ECMO is not beneficial for cardiogenic shock, and the role of VA‐ECMO in cardiogenic shock has been debated. In the ECMO‐CS trial, the ECLS‐SHOCK trial, and subsequent meta‐analyses, VA‐ECMO showed no mortality benefit, regardless of whether cardiac arrest occurred or whether support was initiated early. No benefit was observed in any subgroup. By contrast, in the present cases, patients with infarct‐related cardiac arrest but without cardiogenic shock were suitable candidates for VA‐ECMO to support the intervention, irrespective of the timing of initiation. Our cases differed from the populations studied in ECMO‐CS and ECLS‐SHOCK. Our cases targeted infarct‐related cardiac arrest without cardiogenic shock and with preserved neurological status, and VA‐ECMO was used as a bridge to reperfusion. In contrast, both trials enrolled patients with established cardiogenic shock. This difference in patient selection was associated with a more favorable prognosis. The Society for Cardiovascular Angiography and Interventions (SCAI) shock classification system categorizes cardiogenic shock into five stages (A–E) to assess its severity and predict mortality. These stages range from “at risk” (A) to “extremis” (E), with each stage representing a different level of hemodynamic instability and hypoperfusion [8]. In the SCAI shock classification, cardiac arrest with comatose neurologic status and multiorgan failure was defined as Stage E, impending circulatory collapse; however, cardiac arrest with normal neurologic function did not change the prognosis [9]. The latter group of patients with normal neurological function after cardiac arrest may be candidates for VA‐ECMO to support the intervention. As shown in Figure 6, VA‐ECMO should be considered only in carefully selected patients with infarct‐related cardiac arrest who retain normal neurologic function without cardiogenic shock, where its role is limited to serving as a bridge to reperfusion therapy, similar to its use during cardiac surgery.

Alternative mechanical circulatory support devices differ from those used for VA‐ECMO. The intra‐aortic balloon pump (IABP) provides modest diastolic augmentation and afterload reduction but has consistently failed to demonstrate a survival benefit in large randomized trials such as IABP‐SHOCK II [10]. Although the Impella device offers active left ventricular unloading and forward flow without gas exchange, which may be advantageous in scenarios of predominant LV pump failure, IMPRESS in the Severe Shock trial (NTR3450) showed neutral results compared with IABP [11]. In contrast, VA‐ECMO is unique in its ability to provide full cardiopulmonary support, including oxygenation, which neither IABP nor Impella CP (Cardiac Power) can achieve. Impella CP is a percutaneous microaxial LV assist device that can deliver up to approximately 4 L/min of forward flow via a 14‐Fr pump on a 9‐Fr catheter. In our case, VA‐ECMO was used selectively as a short‐term bridge to reperfusion in infarct‐related cardiac arrest without cardiogenic shock, whereas unloading‐focused devices may be more appropriate for patients with isolated LV dysfunction and preserved oxygenation.

Recently, the DanGer‐SHOCK (Microaxial Flow Pump or Standard Care in Infarct‐Related Cardiogenic Shock) trial evaluated routine use of Impella CP in patients with ST‐segment elevation myocardial infarction complicated by cardiogenic shock. The trial showed that Impella CP plus standard care reduced 180‐day all‐cause mortality by approximately 25% compared with standard care alone [12]. Although the trial used relatively broader diagnostic thresholds for cardiogenic shock (systolic blood pressure < 100 mmHg, lactate > 2.5 mmol/L, and LVEF < 45*%*), routine implantation of Impella CP plus standard care could be beneficial in patients with infarct‐related cardiogenic shock.

**Figure 7:**
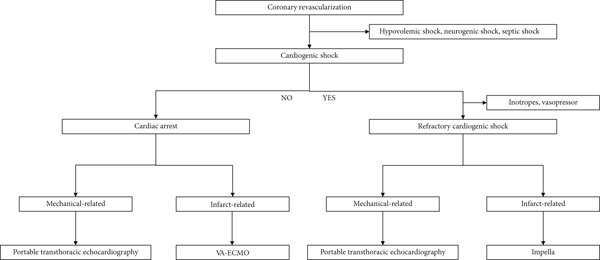
The clinical algorithm of infarct‐related cardiac arrest without cardiogenic shock. In refractory cardiogenic shock, consider Impella as LV unloading; in infarct‐related cardiac arrest without shock, consider VA‐ECMO as a short bridge to reperfusion. VA‐ECMO, venoarterial extracorporeal membrane oxygenation.

Based on these considerations, the algorithm is presented in Figure 7. When cardiogenic shock occurs during coronary revascularization, the first step is to exclude alternative causes, such as hypovolemic shock (e.g., drug‐related hypotension), neurogenic shock (e.g., severe pain), or septic shock. If cardiogenic shock is confirmed, initial management includes administration of inotropes or vasopressors with volume supplementation as needed. In the presence of mechanical complications, such as coronary perforation or tamponade, portable TTE is essential for differentiation. Impella may provide additional support in cases of infarct‐related cardiogenic shock, such as acute myocardial infarction or abrupt coronary closure. Conversely, if cardiac arrest occurs without cardiogenic shock, the underlying cause should be reassessed. Portable TTE is crucial for mechanical causes of arrest, whereas in infarct‐related cardiac arrest, VA‐ECMO can be applied as a bridge therapy to allow intervention completion.

Our report, detailing the cases of three patients, showed that VA‐ECMO could be beneficial for patients with infarct‐related cardiac arrest without cardiogenic shock. Although the role of VA‐ECMO is debatable, these patients could be suitable candidates for VA‐ECMO to support coronary interventions.

## 4. Limitations

This report is limited by its nature as a single‐center case series involving only three patients, which restricts the generalizability of our findings. The favorable outcomes we observed may not necessarily be replicated, particularly in patients with established cardiogenic shock or those with prolonged significant neurologic injury after cardiac arrest. Finally, as this report is a retrospective case series, a causal relationship between VA‐ECMO use and favorable outcomes cannot be established.

## 5. Conclusions

In patients with infarct‐related cardiac arrest without cardiogenic shock, VA‐ECMO may serve as a suitable supportive strategy to facilitate successful coronary revascularization and hemodynamic stabilization. These cases suggest that VA‐ECMO may be considered for carefully selected patients in clinical practice. Larger, prospective studies are warranted further to define its role in this specific patient population.

## Ethics Statement

The Institutional Review Board of Uijeongbu St. Mary′s Hospital, The Catholic University of Korea, reviewed this study and granted an exemption from ethical review (IRB No. UC24ZISI0118).

## Consent

The requirement for informed consent was waived by the Institutional Review Board of Uijeongbu St. Mary′s Hospital, The Catholic University of Korea (IRB No. UC24ZISI0118). All clinical information was deidentified and anonymized.

## Conflicts of Interest

The author declares no conflicts of interest.

## Author Contributions

Seonghyeon Bu: conceptualization, data curation, investigation, methodology, project administration, resources, supervision, validation, visualization, and writing—original draft.

## Funding

The author declares that no financial support was received for the research, authorship, and/or publication of this article.

## Data Availability

The original contributions presented in the study are included in the article; further inquiries can be directed to the corresponding author.
